# Efficient procedures for the numerical simulation of mid-size RNA kinetics

**DOI:** 10.1186/1748-7188-7-24

**Published:** 2012-09-07

**Authors:** Iddo Aviram, Ilia Veltman, Alexander Churkin, Danny Barash

**Affiliations:** 1 Department of Computer Science, Ben-Gurion University, 84105, Beer Sheva, Israel

## Abstract

**Motivation:**

Methods for simulating the kinetic folding of RNAs by numerically solving the chemical master equation have been developed since the late 90's, notably the programs Kinfold and Treekin with Barriers that are available in the Vienna RNA package. Our goal is to formulate extensions to the algorithms used, starting from the Gillespie algorithm, that will allow numerical simulations of mid-size (~ 60–150 nt) RNA kinetics in some practical cases where numerous distributions of folding times are desired. These extensions can contribute to analyses and predictions of RNA folding in biologically significant problems.

**Results:**

By describing in a particular way the reduction of numerical simulations of RNA folding kinetics into the Gillespie stochastic simulation algorithm for chemical reactions, it is possible to formulate extensions to the basic algorithm that will exploit memoization and parallelism for efficient computations. These can be used to advance forward from the small examples demonstrated to larger examples of biological interest.

**Software:**

The implementation that is described and used for the Gillespie algorithm is freely available by contacting the authors, noting that the efficient procedures suggested may also be applicable along with Vienna's Kinfold.

## Background

The RNA molecule, once considered as an intermediate step between DNA and proteins, has drawn much attention in recent years. Discoveries relating to its unique capabilities to prominently participate in gene regulation have motivated even more the concerted efforts to understand its folding and structural arrangement at several levels, both at the level of tertiary structure and that of secondary structure. The functional form of single stranded RNA molecules frequently requires a specific tertiary structure, but the scaffold for this structure is provided by secondary structural elements which are hydrogen bonds within the molecule. The four building blocks of RNAs are A,C,G,U and the base pairings among them form the secondary structure. This leads to several recognizable "domains" of secondary structure like hairpin loops, bulges and internal loops. Although the functional role of the RNA molecule in more detail is related to its three-dimensional structure, the RNA secondary structure is experimentally accessible and in many interesting cases may contain substantial important information to shed light on the relationship between structure and function. In general, RNA folding is thought to be hierarchical in nature [1,2], whereby a stable secondary structure forms first and subsequently there is a refinement to the tertiary fold. Thus, RNA conformational rearrangements that will be mentioned in the discussion can often be studied by examining their secondary structure, while keeping in mind the importance of tertiary structure.

When attempting to simulate the complete folding event, the time needed to reach equilibrium can become very long and it is computationally too expensive to consider the kinetics of the tertiary structure by using a molecular dynamics approach. Therefore, beyond the static view of RNA folding using energy minimization methods to predict the final state of the folding, a time-dependent view is desired in order to extract information on the folding kinetics. To do so practically, it is imperative to simulate the complete folding event at the level of RNA secondary structure. For that, the chemical master equation can be solved numerically by a stochastic simulation algorithm, as was first shown in that context in [3,4]. Potentially, important information can be extracted from such a simulation that considers the suboptimal solutions, which were dealt with before in refs [5,6] in an informative manner. The motivation and importance from the biochemical perspective for this type of computational simulations, also describing the experimental observables that can be extracted from the calculations, can be found in [7]. A recent comprehensive review of the field is available in [8]. Other computational simulation approaches for RNA kinetics besides Kinfold [4] that are beyond the regime of the mid-size RNA kinetics described herein include RNAkinetics [9], Kinefold [10], and Kinwalker [11] for large RNAs. To further motivate the time-dependent view, it was shown in [12] that RNA genes not only encode information about their functional structure, but also on their co-transcriptional folding pathway (and, e.g. transient structures). More recently, kinetically trapped RNA secondary structures were thermodynamically analysed in [13] and an efficient method for computing folding pathways between RNA secondary structures was developed in [14] that follows the work of [15] on determining an optimal folding pathway and barrier energies introduced in [4,16]. For an overview on RNA folding kinetics and the importance of RNA folding intermediates, some recent review articles are available in [17-21].

## Methods

The complete folding event is governed by the chemical master equation [8]. In order to introduce the concept behind the reduction of the time-dependent RNA folding problem to that of stochastic chemical kinetics describing the time evolution of a well-stirred chemically reacting system, the Appendix follows closely references [22,23] in summarizing the formulation leading to Gillespie's Stochastic Simulation Algorithm (SSA).

Our goal is to model the problem of RNA secondary structure folding in such a way that it can be reduced to the algorithm with the pseudocode given in the Appendix. Therefore, we will describe a reduction into the Stochastic Simulation Algorithm (SSA). The rationale behind this way of formulating the problem is that after the reduction it becomes easier to devise an efficient version of the SSA for RNA folding kinetics, with multiple runs performed in parallel (see SSA version II and discussion thereafter).

Using the Vienna's notations as can be found in [4,24], the RNA sequence in time *t* will be represented as two strings. Both are of the size of the RNA sequence. One is over the character set {A,U,G,C} also providing what the order of the nucleotides is. It will be called from here on the 'sequence string'. The other is a string of balanced parentheses over the character set {., (,)}, known as "dot-bracket" notation, describing the secondary structure of the RNA sequence (dot means no base-pairing, and each open and close parentheses represent a base pairing). It will be named here the 'structure string'. We shall notice that while the former does not change over time, the latter does.

In attempting to simulate over time the secondary-structure changes of a certain RNA sequence, let us denote $S_{R}\left(t\right)$ as the random variable that contains what is the structure string of the RNA structure at time t, when the sequence string is known to be R. In the settings of this simulation, $S_{R}\left(0\right)$is set to be the string "…,⋯,…", which is the initial folding open state without any base pairings. Our goal is to predict what $S_{R}\left(t\right)$ is for some parameter *t*. In particular, we would like to predict how much time it will take for an RNA sequence to fold into its 'optimal' state, defined as the structure whose Gibbs free energy is minimal. To formalize that, we will denote the optimal structure for the sequence R as Op(R). Thus, our simulation goal is to find the smallest *t* for which$S_{R}\left(t\right)=Op\left(R\right)$.

Having defined our goal, we will introduce some more notations to explain the reduction. $F_{R}=\left\{s_{R}^{1},s_{R}^{2},\ldots ,s_{R}^{M\left(R\right)}\right\}$ will be the finite set (whose size is denoted by M(R)) of the feasible structure strings for the sequence string R, feasible meaning taking into account biological constraints. Now, for some 1 ≤ i ≤ M(R), a single step move of $s_{R}^{i}$ is a structure string $s_{R}^{j}\in F_{R}$ such that $s_{R}^{j}$ and $s_{R}^{i}$ differ only by omitting a pair of parentheses, adding a pair, or flipping a pair in the way that is well described in [4]. We will define the neighbourhood of $s_{R}^{i}$, denoted $N_{R}^{i}=\left\{s_{R}^{i_{1}},s_{R}^{i_{2}},\ldots ,s_{R}^{i_{L}}\right\}$, as the subset of $F_{R}$ which can be reached from $s_{R}^{i}$within a single step, unless $s_{R}^{i}=OP\left(R\right)$, in which case we will define $N_{R}^{i}$ to be an empty set.

Gillespie's SSA deals with simulations of reactions of a system [22,23]. A possible reaction from molecule of structure $s_{a}$ into molecule of structure $s_{b}$ is denoted $s_{a}\to s_{b}$. We will define a shortened notation for a set of possible reactions:

(1) $$s_{a}\to s_{B}=\left\{{s_{b}}_{1},{s_{b}}_{2},\ldots ,{s_{b}}_{p}\right\}=s_{a}\to s_{b1},s_{a}\to s_{b2},\ldots ,s_{a}\to s_{\mathrm{bp}}\text{.}$$

The reduction of the input is done by treating single-step moves of the RNA structure as 'possible reactions'. Using the given notations, we will obtain that the total possible moves are: $s_{R}^{1}\to N_{R}^{1}s_{R}^{2}\to N_{R}^{2},\ldots ,s_{R}^{M\left(R\right)}\to N_{R}^{M\left(R\right)}$. But, since the simulation is at a specific time at state $s_{R}^{i}$for some 1 ≤ i ≤ M(R), we are only left with the feasible moves of $s_{R}^{i}\to N_{R}^{i}$. These moves will constitute the reaction set of Gillespie's algorithm. Now, the probability factor of each move to occur in a time of Δt is calculated according to the Gibbs free energy considerations using the program RNAeval available in the Vienna RNA package, along with a stochastic Monte Carlo feature. The equation we used in our implementation is the one based on the simulated annealing approach [25] and known as the Metropolis [26] step, which is:

(2) $$a_{\mathrm{ij}}=\{\begin{array}{c} \exp\left(-\Delta G/RT\right),if\Delta G\geq 0,\\ 1,otherwise\text{.} \end{array}$$

$\text{A}=\left[a_{\mathrm{ij}}\right]$ is the transition probability matrix, with $\Delta G=G_{j}^{0}-G_{i}^{0}$ when considering the rate of a transition to *j*, being at *i*. $G_{i}^{0}$is the Gibbs free energy of *i* for each secondary structure *i* for the SSA algorithm version 1 below; for the SSA algorithm version 2 that follows, it is the sum of the free energies over an n-tuple of secondary structures. We shall note that in our implementation, we used Vienna's own program called RNAeval to get the Gibbs free energy values for the RNA structures. In addition, similar to Vienna's Kinfold, a Kawasaki step can be used instead of the Metropolis step in the equation above. We can observe that we now have a proper input problem that fits Gillespie's SSA algorithm. Thus, we can use the following algorithm:

### SSA for RNA folding, version I – simulating one RNA-structure fold

1. The current structure is $s_{R}^{i}$ for some 1 ≤ i ≤ M(R). While stopping condition $s_{R}^{i}$ equals to Op(R) is not met:

2. Calculate $N_{R}^{i}$.

3. Evaluate for each member of $N_{R}^{i}$ its probability factor, and the total sum of the factors. We define $a_{R}^{k}$ as the probability factor of $s_{R}^{i}\to s_{R}^{k}$ if $s_{R}^{k}\in N_{R}^{i}$ and 0 otherwise. We will also denote $a_{\mathrm{sum}}$ as the total sum of all the factors.

4. Draw two independent uniform (0,1) random numbers: ξ_1_ and ξ_2_.

5. Set j to be the smallest integer satisfying $\sum_{k=1}^{j}a_{R}^{k}>\xi_{1}a_{\mathrm{sum}}\text{.}$

6. Set $\tau =\frac{\ln\left(1/\xi_{2}\right)}{a_{\mathrm{sum}}}\text{.}$

7. Set the current structure to be $s_{R}^{j}$, and the time to be (t + τ). Return to step 1.

At this point, a beneficial observation is that we can actually expand the model to run this way many simulations simultaneously. If we have different RNA sequences with the sequence strings of $R_{1},R_{2},R_{3},\ldots ,R_{n}$ as in all possible single point mutations, we will have the possible moves of $s_{R1}^{1}\to N_{R1}^{1},s_{R1}^{2}\to N_{R1}^{2},\ldots ,s_{R1}^{M\left(R1\right)}\to N_{R1}^{M\left(R1\right)}\text{,}$$s_{R2}^{1}\to N_{R2}^{1},s_{R2}^{2}\to N_{R2}^{2},\ldots ,s_{R2}^{M\left(R2\right)}\to N_{R2}^{M\left(R2\right)},\ldots ,s_{\mathrm{Rn}}^{1}\to N_{\mathrm{Rn}}^{1},s_{\mathrm{Rn}}^{2}\to A_{\mathrm{Rn}}^{2},\ldots \text{,}$. Let $s_{R1}^{i1},s_{R2}^{i2},\ldots ,s_{\mathrm{Rn}}^{\mathrm{in}}$ be the current states of the n RNA sequences, then we are left with the feasible moves of $s_{R1}^{i1}\to N_{R1}^{i1},s_{R2}^{i2}\to N_{R2}^{i2},\ldots ,s_{\mathrm{Rn}}^{\mathrm{in}}\to N_{\mathrm{Rn}}^{\mathrm{in}}$.

Using these formulations, we suggest a somewhat optimized and generalized variation of the aforementioned algorithm. In the following version, the indices *i* and *j* will not anymore correspond directly to an explicit $\left[a_{\mathrm{ij}}\right]$ matrix instance of the Metropolis step's equation. Instead, although we still are doing a Metropolis step, it will correspond to a much larger matrix which is defined only implicitly.

### SSA for RNA folding, version II – simulating numerous RNA-structure folds

1. The initial structure array is $S_{\mathrm{arr}}=<s_{R_{1}}^{i_{1}},s_{R_{2}}^{i_{2}},s_{R_{3}}^{i_{3}},\ldots ,s_{R_{n}}^{i_{n}}>$.

2. Calculate$N_{\mathrm{arr}}=<N_{R_{1}}^{i_{1}},N_{R_{2}}^{i_{2}},N_{R_{3}}^{i_{3}},\ldots ,N_{R_{n}}^{i_{n}}>$.

3. Evaluate for each member of $N_{R_{j}}^{i_{j}}\in N_{\mathrm{arr}}$ its probability factor, and the total sum of the factors. We denote by $a_{R_{i}}^{k_{i}}$ as the factor related to the k'th member of $N_{R_{j}}^{i_{j}}$, and $a_{\mathrm{sum}}$ the total sum of all the factors related to $N_{R_{1}}^{i_{1}},N_{R_{2}}^{i_{2}},N_{R_{3}}^{i_{3}},\ldots ,N_{R_{n}}^{i_{n}}\text{.}$ Having all the factors ordered by some total order, we will obtain a series $\left(a_{k}\right)$. Each member of $\left(a_{k}\right)$ corresponds to a specific member of $N_{\mathrm{arr}}$, and we will denote this mapping to the corresponding $N_{\mathrm{arr}}$ indices by $\left(m_{k}\right)$.

4. While $S_{\mathrm{arr}}$ is not equal to$<Op\left(R_{1}\right),Op\left(R_{2}\right),Op\left(R_{3}\right),\ldots ,Op\left(R_{n}\right)>$:

5. Draw two independent uniform (0,1) random numbers: ξ_1_ and ξ_2_.

6. Set j to be the smallest integer satisfying $\sum_{k=1}^{j}a_{k}>\xi_{1}a_{\mathrm{sum}}$. We shall denote j* and k* as the indices for which $a_{j}=a_{k*}^{j*}\text{.}$

7. Set $\tau =\frac{\ln\left(1/\xi_{2}\right)}{a_{\mathrm{sum}}}\text{.}$

8. Set the $m_{j}$'th component of $S_{\mathrm{arr}}$ to be $s_{R*}^{j*}$, the time to be (t + τ).

9. Recalculate the $m_{j}$'th neighborhood (with its corresponding factors, $a_{R_{i}}$), and update $N_{\mathrm{arr}}$ as well as $\left(a_{k}\right)$ and $a_{\mathrm{sum}}$. Return to step 4.

Repetitions of the same experiment, i.e. setting $R_{1}=R_{2}=R_{3}=\ldots =R_{n}$, are useful to estimate the smallest *t* for which $S_{R}\left(t\right)=Op\left(R\right)$. This information is, as told at first, what we sought for, and what we actually implemented. In this special case, memoization might be useful in calculating the expensive step 9 above. Because folding patterns tend to be very repetitive, even a relatively small-sized memoization might save a significant amount of computation time. We implemented a simple memoization and were able to run it on short sequences (up to 40 nt). We measured running times with a memoization that memorizes the neighborhoods of 10,000 different structures. The allocated RAM space was large enough not to require swapping. We obtained a significant speedup: 43 seconds with memoization vs. 684 seconds without memoization for a sequence of size 35, with 100 simultaneous simulations, and approximately 6 seconds with memoization against 60 seconds without memoization for a sequence of size 20, with 1000 simultaneous simulations. When implementing memoization, keeping in memory all neighborhood sets possible is not feasible since their number grows too fast in respect to the size of the sequence. But because most of the transitions occur in the basins of the local minima, in terms of the energy values of the structures, keeping a fixed number of sets may suffice to decrease the computation time. We suggest the use of a cache-like LRU (least recently used) algorithm as mentioned above for deciding which information is likely to be re-usable among all neighboring sets and probabilities ever computed during a run. In addition, interesting ideas can be developed to make the memoization more efficient along the lines of calculating useful measures in order to assess the "folding progress" of all molecules. Since folding times may well be distributed over several orders of magnitude, one may want to let those molecules that have "fallen behind" given some time to catch up, such that all molecules fold at approximately the same speed without large time deviations that are problematic because of having to wait for the slowest molecules to terminate. A simple and practical candidate for such a measure is the base pair distance between the starting and stopping structures. In addition, we claim that the strategy outlined in the SSA for RNA folding, version II above is better tailored for biological problems in which it is not necessary to wait for all molecules to reach their target structure. For such type of problems, there are several advantages to our approach. First, when a particular molecule gets folded, it frees its memoization resources and also reduces the size of the probability space in the sense that it makes the transitions of the rest of the molecules more probable to be the next to occur.

Aside of memoization, an advantage of the approach of the SSA for RNA folding version II presented above over a repetition of single structure simulations is that we can stop the simulation after an elapsed simulation time τ, and extract the folding time of all the experiments that were already folded in time which is the most τ. Moreover, taking this approach, no single long-lasting simulation can delay the intermediate results of the overall run. It is well on our interest that the molecules that are last to be folded will not constitute a bottleneck for the whole computation. In our settings, if we could use an anytime computation approach in which the probability is revealed gradually, what we might give up by not waiting for the last molecules to terminate is just the extent of a long probability tail. If the whole computation is stopped before all molecules have terminated their folding, it may again be useful to calculate their base pair distances to the stopping structures in order to predict the amount of error by not letting all molecules terminate their folding. For some problems the error might be small enough or the computation can be resumed some more until the approximation is satisfactory for the particular problem's needs. It should also be noted that if the sequence strings $R_{1},R_{2},R_{3},\ldots ,R_{n}$ are different mutants of the same wildtype then the ideas discussed above can still be used to considerably reduce the computation time.

## Results

Here below, we demonstrate our SSA implementation on two "toy problem" examples. Our program that we have been developing is similar in style to Vienna's Kinfold [4], but has been built in principle to have the ability of exploiting memoization and efficiency considerations as proposed above.

**Figure 1:**
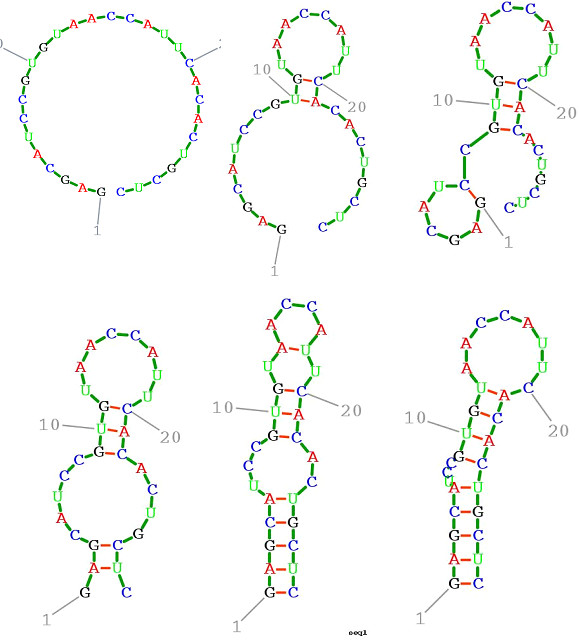
**Ex1: Starting from open chain (left), six stages during the folding.** SSA version I, sequence ‘GAGCAUCCGUGUAACCAUUCACACUGCUC ' is used.

**Figure 2:**
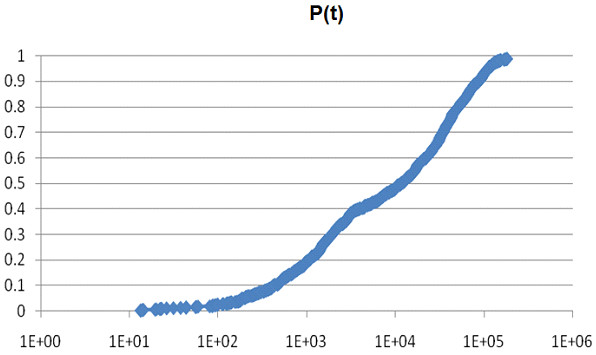
Ex1: Distribution of folding times.

**Figure 3:**
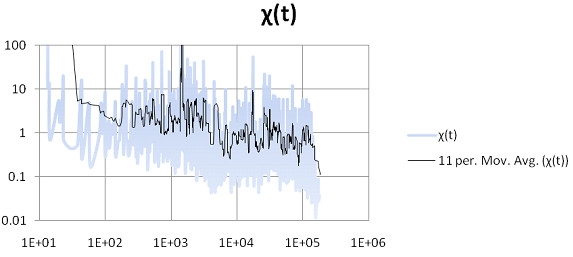
Ex1: Time evolution of the folding characteristic.

**Figure 4:**
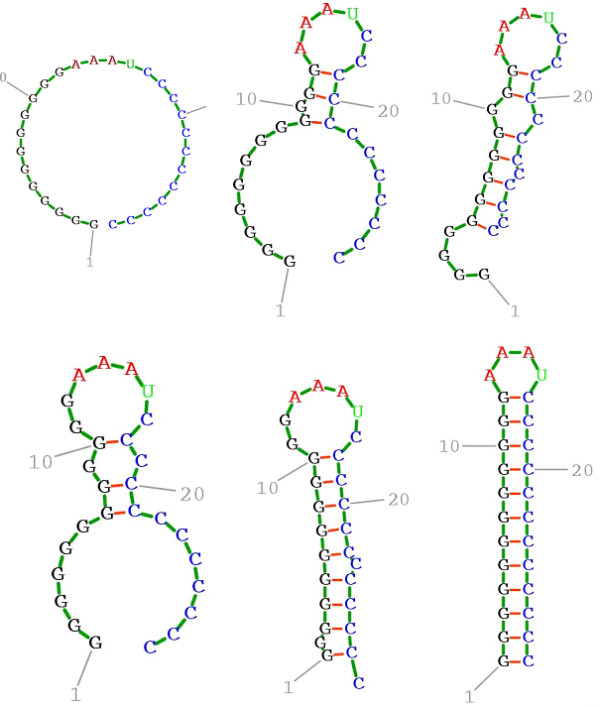
**Ex2: Starting from open chain (left), six stages during the folding.** SSA version I, sequence `GGGGGGGGGGGGAAAUCCCCCCCCCCCC ' is used.

**Figure 5:**
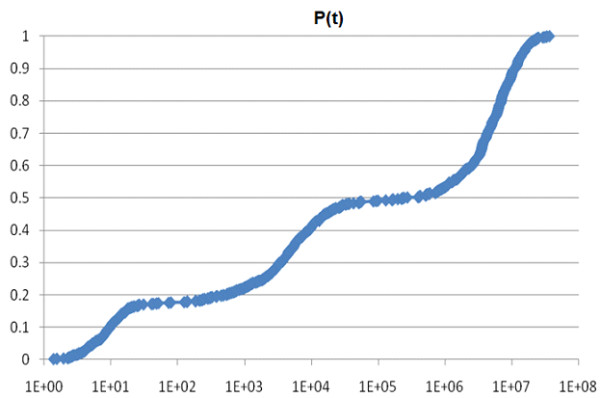
Ex2: Distribution of folding times.

**Figure 6:**
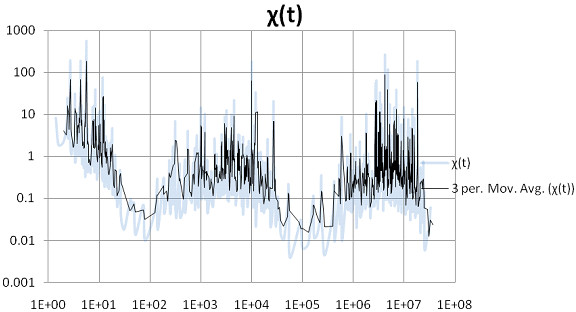
Ex2: Time evolution of the folding characteristic.

Before examining Figures 1, 2, 3, 4, 5, 6, the two respective sequences for Example 1 and Example 2 are given below. Next, for each of the two examples, the RNA secondary structures in 5 different stages are drawn, after the drawing of the open chain at the very left of Figures 1 and 4, respectively. The main analysis plots of the time-dependent simulations are the distribution of folding times $P\left(t\right)$in Figures 2 and 5, and the folding characteristic in Figures 3 and 6. The distribution of folding times is the fraction of folding trajectories that reached the mean free energy structure plotted on a logarithmic time scale, with the time units arbitrary. The plot was generated after collecting 1000 points. The folding characteristic is given by $t*P'\left(t\right)/P\left(t\right)$and distinctive humps on this figure correspond to different folding paths. These figures, namely the distribution of folding times and the folding characteristic, are well described in Flamm et al. 2000 [4] and are given here for consistency with the aforementioned paper that describes Kinfold. In our two examples and our SSA implementation, we can clearly see that the sequence of Example 1 in Figure 3 evolves much smoother with no humps in the folding characteristic compared to the sequence of Example 2 in Figure 6 that displays 3 humps in the folding characteristic. Next, a representative output of the evolved RNA secondary structure in "dot-bracket" notation is outlined in two tables, for Example 1 in Additional file 1: Table S1 and for Example 2 in Additional file 2: Table S2, respectively, with the 5 underlined structures for each example drawn in Figures 1 and 4, respectively. 

The sequences used for illustration in Figures 1, 2, 3, 4, 5, 6 are:

Example 1: SSA version I, R = ` GAGCAUCCGUGUAACCAUUCACACUGCUC '

Example 2: SSA version I, R = ` GGGGGGGGGGGGAAAUCCCCCCCCCCCC '

## Discussion

As a possible application of biological significance, the time-dependent approach discussed above is suggested for beneficial use in the problem of deleterious mutation prediction. To elaborate on this problem, a common way to detect deleterious mutations in the secondary structure of RNAs is to look for mutations that may cause a conformational rearrangement to occur. It was noted in [27] that there is some probability that even a single mutation can substantially alter the RNA secondary structure. Experimentally, this was observed in the spliced leader of *Leptomonas collosoma*[28], in RNA viruses [29,30], and in some other biological systems. Another very recent finding of biological importance is the existence of disease-associated Single Nucleotide Polymorphisms (SNPs) called "RiboSNitches" that have an RNA secondary structural consequence that results in a disease phenotype [31]. Computationally, even before the added motivation as a consequence of the latter disease related finding, this gave rise to a procedure for detecting deleterious mutations using RNA folding predictions numerous times [32]. Each time, relevant programs from an energy minimization package such as RNAfold from the Vienna RNA package [24,33] or Zuker's mfold [34,35] can be used. In these packages, expanded energy rules [36] that were derived from an independent set of experiments are incorporated into the folding prediction algorithm. While the folding prediction problem described above is the most fundamental problem in RNA bioinformatics, the RNA mutation prediction problem is a sub-problem that uses the former multiple times, for various mutation combinations. Historically, initial works for the mutation prediction problem can be traced back to [37,38] and have been substantially revived in [32,39]. The first publicly available program for the RNA mutation prediction problem that takes into account only single-point mutation predictions was called RNAMute [40,41]. It uses the Vienna RNA package in its core. Subsequently, a web server dealing with similar issues was put forth called RDMAS [42]. There are also some computationally challenging issues in the mutation prediction problem [43], mainly in the generalization to multiple-point mutations that can become computationally heavy if a 'brute-force' strategy of calculating all possible mutations is used without devising any unique approach. There have been various suggestions on how to reduce the number of mutations simulated or make the computations more efficient, for example [44-46]. In general, neither the original RNAMute [41] nor RDMAS [42] can handle multiple-point mutations. Consequently, RNAMute [41] was extended to MultiRNAMute [44] and based on the approach of [45], the web servers RNAmutants [47] and later corRna [48] were developed. A web server for MultiRNAMute was worked out in [49]. There is, however, one common feature that should be taken into account when considering all of the programs dealing with RNA deleterious mutation predictions. Because several single point mutations inserted to the wildtype sequence can bring about to the same secondary structure, oftentimes there is a degeneracy in the mutations that is needed to be addressed. Any mutation prediction method for the purpose of conformational rearrangement in the secondary structure should therefore aim to report in each step (i.e., one-mutation, two-mutations, etc.) several mutation possibilities, not only a single one. If the method only reports a single possible mutation in each step, it probably means that a computational efficiency consideration was taken that may neglect many good candidate mutations that are conformationally rearranging just as well and will lead to the same secondary structure. Therefore, in order to fundamentally solve the degeneracy of mutations leading to the same structure, we suggest to perform for each one a time-dependent calculation and check how smooth the folding occurs in time, to discriminate those mutation candidates that get stuck in a local optimum for a while during the folding in time. This is quite an intensive calculation for sequences that are beyond "toy problems", leading to a computational challenge from the numerical standpoint. It is also of considerable interest to check whether there is a correlation between the kinetic calculation and the static information obtained by performing energy minimization without taking into account what happens during the folding event. In order not to lose reliability, we suggest to consider all single point mutation combinations, and decide which one is the most likely to occur without getting trapped in a local minimum by using a time-dependent approach.

**Figure 7:**
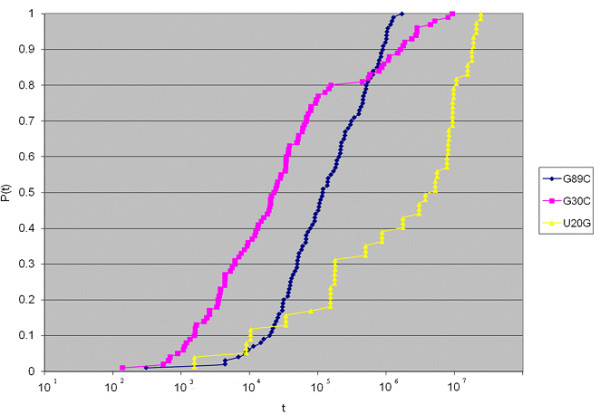
Demonstrating feasibility of the time-dependent approach in RNA deleterious mutation prediction.

In Figure 7, the idea of using a time-dependent approach for RNA deleterious mutation prediction is exemplified on a segment taken from an HCV IRES within the 5' UTR, for which experimental results are already known and the wildtype structure is well predicted by energy minimization. It was found in an experiment on this segment [29], which is notably located far away from the well-known pseudoknot of the IRES and its folding is well predicted by mfold and the Vienna RNA package when compared to the experimental result, that a single point mutation will cause a dramatic reduction in translation initiation. With RNAMute [41] it is possible to capture this mutation inside a list of about 60 other selected single point mutations that can potentially induce a conformational rearrangement, all resulting in a common or very similar secondary structure when checked with mfold or Vienna's RNAfold. Consequently, the question that a potential rational design experiment might want to address is which mutation will likely show the most pronounced affect, assuming that a reduction in translation initiation correlates with a smooth transition from the wildtype structure to a conformationally rearranging one without getting trapped in local minima. Figure 7 displays the result of Kinfold [4] for 3 mutations from the list of 60 candidate mutations, providing an indication of how smooth is the folding for each mutant. For mutations G89C and G30C, about 100 points were collected when generating the distribution of folding times $P\left(t\right)$, which all together took from several hours to more than a day, and for the mutation U20G only 20 points were collected in the course of more than 2–3 days. From the one hand, the feasibility of a time-dependent simulation for such a sequence that is biologically significant and in the range of mid-size RNA (60–150 nt long) is qualitatively demonstrated in Figure 7 for a small sample of points. From the other hand, it is clearly evident that computational strategies to reduce the heavy computation time, as proposed here with the SSA for RNA folding version II, can be of benefit. 

## Conclusions

The significance of the initial development of efficient procedures described here can be divided into several items. First, the time-dependent folding is what takes place within the RNA molecule, and the static view of RNA structure only at the beginning and end may not be sufficient or complete in many cases. Experimental approaches to measure folding kinetics in detail, such as temperature jump experiments or single molecular methods [7], can be employed to check the computational model and predictions, in turn, can be pivotal to RNA rational design. Developing efficient numerical methods for the time-dependent folding simulation is therefore, by itself, an important goal. Here, we embarked on the stochastic approach, noting that if at all possible to achieve with practical computation time then one should definitely consider deterministic approaches [50] for the simulations of biologically relevant examples. Another direction for reducing computational cost is by an efficient exploration of discrete energy landscapes, which was developed in a recent work [51] by introducing a sampling method that allows for a fast yet accurate estimation of the transition probabilities between macrostates when coarse graining of the state space is used [4,16,50,52,53]. Second, a time-dependent approach to contribute in deleterious mutation prediction is suggested, which is still an open problem of considerable biological interest in a variety of RNA systems. For example, point mutations performed on an RNA virus such as HCV can alter virus replication [30] or lead to a dramatic reduction in translation initiation [29]. Development of efficient time-dependent simulations can well assist from the predictive standpoint in such efforts.

## Appendix

The probability of a reaction $R_{k}$to occur in an infinitesimal time interval [t, t + dt) will be denoted by $a_{k}\left(X\left(t\right)\right)dt$ known as the propensity function. Applying the law of total probability, one can obtain:

(3) $$\begin{array}{l} P(x,t+dt)=\left(1-\sum_{j=1}^{M}a_{j}\left(x\right)dt\right)P(x,t)+\sum_{j=1}^{M}a_{j}\left(x-v_{j}\right)dtP(x-v_{j},t)\\ \Leftrightarrow \frac{P\left(x,t+dt\right)-P\left(x,t\right)}{dt}=\sum_{j=1}^{M}(a_{j}\left(x-v_{j}\right)P(x-v_{j},t)-a_{j}P(x,t))\\ \overset{dt\to 0}{\Rightarrow }\frac{dP\left(x,t\right)}{dt}=\sum_{j=1}^{M}(a_{j}\left(x-v_{j}\right)P(x-v_{j},t)-a_{j}P(x,t)) \end{array}$$

The last equation is known as the chemical master equation. It is a set of linear ordinary differential equations (ODEs), one ODE for each possible state of the system. Solution of each of the equations at time t is a real number giving the probability of the system being in that particular state at time t. X(t) is the state vector, X(0) is the initial condition, and the quantity P(x,t) is the probability that X(t) = x. The inputs to the equation are the chemical reactions and their propensity function $a_{k}\left(X\left(t\right)\right)$. An illustrative example of how this equation is applied for studying the Michaelis-Menten model system is available in [23]. In order to solve the chemical master equation for practical cases, a stochastic simulation algorithm was devised, also known as the Gillespie algorithm [22], by simulating the changes in the system as they evolve in time. Because single steps are treated, the following quantities are introduced:

p(τ,j|x,t) is the probability that reaction j happens in the time interval [t,t + τ] given that X(t) = x.

p(τ,0|x,t) is the probability that no reaction happens in the time interval [t,t + τ] given that X(t) = x.

It is assumed that p(τ,0|x,t) and p(dτ,0|x,t + τ) are independent. It follows that: $\begin{array}{l} P(\tau +d\tau ,0|x,t)=P(\tau ,0|x,t)*P(d\tau ,0|x,t+\tau )=P(\tau ,0|x,t)\left(1-\sum_{k=1}^{M}a_{k}\left(x\right)d\tau \right)\\ \Leftrightarrow \frac{P\left(\tau +d\tau ,0|x,t\right)-P\left(\tau ,0|x,t\right)}{d\tau }=-a_{\mathrm{sum}}(x)P(\tau ,0|x,t),wherea_{\mathrm{sum}}=\sum_{k=1}^{M}a_{k}\left(x\right)\\ \overset{dt\to 0}{\Rightarrow }P(\tau ,0|x,t)=e^{-a_{\mathrm{sum}}\left(x\right)\tau } \end{array}$And, since: $P\left(\tau ,j|x,t\right)=P\left(\tau ,0|x,t\right)a_{j}\left(x\right)d\tau$Then $\begin{array}{l} P(\tau ,j|x,t)=a_{j}(x)e^{-a_{\mathrm{sum}}\left(x\right)\tau }\\ \Leftrightarrow P(\tau ,j|x,t)=\frac{a_{j}\left(x\right)}{a_{\mathrm{sum}}\left(x\right)}a_{\mathrm{sum}}(x)e^{-a_{\mathrm{sum}}\left(x\right)\tau } \end{array}$The last equation shows that $P\left(\tau ,j|x,t\right)$ can be written as the product of two individual density functions:

Next reaction index $a_{j}\left(x\right)/a_{\mathrm{sum}}\left(x\right)$ corresponds to a discrete random variable: pick one of the reactions with the rule that the chance of picking the *j*th reaction is proportional to the propensity function $a_{j}\left(x\right)$.

Time until next reaction $a_{\mathrm{sum}}\left(x\right)e^{-a_{\mathrm{sum}}\left(x\right)\tau }$ is the density function for a continuous random variable with an exponential distribution. These exponential random variables arise universally in descriptions of the time elapsing between unpredictable events.

The resulting algorithm for solving the master equation using the Stochastic Simulation Algorithm (SSA, or the Gillespie algorithm) can now be described with the following pseudocode:

## SSA for chemical reactions

1. Evaluate ${\left\{a_{k}\left(x\left(t\right)\right)\right\}}_{k=1}^{M}$ and $a_{\mathrm{sum}}=\sum_{k=1}^{M}a_{k}\left(x\left(t\right)\right)\text{.}$

2. Draw two independent uniform (0,1) random numbers: ξ_1_ and ξ_2_.

3. Set j to be the smallest integer satisfying $\sum_{k=1}^{M}a_{k}\left(x\left(t\right)\right)>\xi_{1}a_{\mathrm{sum}}\left(x\left(t\right)\right)\text{.}$

4. Set $\tau =\frac{\ln\left(1/\xi_{2}\right)}{a_{\mathrm{sum}}\left(x\left(t\right)\right)}$

5. Set x(t + τ) = x(t) + ν_j_ and update t to t + τ

6. If no stopping condition is met, return to step 1.

This is the basic algorithm for simulating chemical reactions that is described in more detail in [22,23], and has been used as well for simulating RNA folding kinetics [4].

## Competing interests

The authors declare that they have no competing interests.

## Authors' contributions

IA and IV and AC worked on the software design, carried out development and implementation, and participated in drafting the manuscript. DB conceived the study, coordinated the software design and drafted the manuscript. All authors read and approved the final manuscript.

## Supplementary Material

Additional file 1 **Table S1.**Ex1: Output illustration of the evolved RNA secondary structure. The list is in "dot-bracket" notation. Energies are in kcal/mol and time units are arbitrary. The five underlined secondary structures of Ex1 are drawn in Figure 1. Click here for file

Additional file 2 **Table S2.**Ex2: Output illustration of the evolved RNA secondary structure. The list is in "dot-bracket" notation. Energies are in kcal/mol and time units are arbitrary. The five underlined secondary structures of Ex2 are drawn in Figure 4. Click here for file
