# Supplementary material for: Efficient procedures for the numerical simulation of mid-size RNA kinetics
Source: Algorithms Mol Biol. 2012 Sep 7;7:24. doi: 10.1186/1748-7188-7-24 (PMC3463434; doi:10.1186/1748-7188-7-24)
Supplement: Additional file 1 — Table S1.Ex1: Output illustration of the evolved RNA secondary structure. The list is in "dot-bracket" notation. Energies are in kcal/mol and time units are arbitrary. The five underlined secondary structures of Ex1 are drawn in Figure 1. [file 1748-7188-7-24-S1.doc]

**Sequence** **Energy** **Time**

............................. 0.00 12.559

..........(........)......... 2.40 34.354

.........((........))........ 1.80 34.419

........(((........)))....... -2.20 34.848

........(((.(.....))))....... 0.40 54.385

........(((........)))....... -2.20 54.718

........(((.(....).)))....... 0.10 58.404

........(((........)))....... -2.20 58.617

(.....).(((........)))....... 0.80 79.648

........(((........)))....... -2.20 80.366

(.....).(((........)))....... 0.80 89.545

........(((........)))....... -2.20 90.000

...

...

...

........(((.(.....))))....... 0.40 761.899

........(((........)))....... -2.20 762.014

..(.....(((........)))....).. 1.30 774.410

.((.....(((........)))....)). 0.60 774.425

(((.....(((........)))....))) -1.80 774.701

((((....(((........)))...)))) -5.40 775.965

(((((...(((........)))..))))) -7.20 776.267

((((((..(((........)))).))))) -4.20 776.836

(((((...(((........)))..))))) -7.20 777.103

.((((...(((........)))..)))). -4.80 811.454

(((((...(((........)))..))))) -7.20 813.416

.((((...(((........)))..)))). -4.80 840.621

(((((...(((........)))..))))) -7.20 840.774

...

...

...

((((....(((........)))...)))) -5.40 92586.614

(((((...(((........)))..))))) -7.20 92587.601

(((((...(((.(.....))))..))))) -4.60 92590.158

(((((...(((........)))..))))) -7.20 92590.580

((((....(((........)))...)))) -5.40 92593.528

(((((...(((........)))..))))) -7.20 92593.535

(((((...(((.(....).)))..))))) -4.90 92594.266

(((((...(((........)))..))))) -7.20 92595.403

(((((...(((........))..)))))) -1.00 92600.441

(((((...(((........)..))))))) -1.10 92601.026

(((((...((............))))))) -3.00 92602.110

(((((...(((..........)))))))) -6.00 92604.991

(((((...((((........))))))))) -7.50 92606.615
