# Supplementary material for: Efficient procedures for the numerical simulation of mid-size RNA kinetics
Source: Algorithms Mol Biol. 2012 Sep 7;7:24. doi: 10.1186/1748-7188-7-24 (PMC3463434; doi:10.1186/1748-7188-7-24)
Supplement: Additional file 2 — Table S2.Ex2: Output illustration of the evolved RNA secondary structure. The list is in "dot-bracket" notation. Energies are in kcal/mol and time units are arbitrary. The five underlined secondary structures of Ex2 are drawn in Figure 4. [file 1748-7188-7-24-S2.doc]

**Sequence** **Energy** **Time**

............................ 0.00 0.000

..........(.......)......... 2.20 2.842

..........(........)........ 1.90 2.874

..........((......))........ -0.20 3.183

........(.((......)))....... 0.30 3.295

.......((.((......))).)..... 0.80 3.431

......(((.((......))).)).... -2.50 3.535

.....((((.((......))).)).).. -2.00 3.731

.....((((.((......)).))).).. -5.90 3.749

.....((((.((......)).)).)).. -5.90 3.822

....(((((.((......)).)).)).) -4.60 3.962

.....((((.((......)).)).)).. -5.90 4.314

....(((((.((......)).)).)).) -4.60 4.375

.....((((.((......)).)).)).. -5.90 4.603

...(.((((.((......)).)).))). -5.40 5.225

...(.((((.(........).)).))). -3.30 5.576

...(.((((.(........).).)))). -3.30 5.607

...(.((((((........))).)))). -10.30 5.815

...(.((((((........)).))))). -10.30 5.964

...(.((((((........)).)))).) -9.60 5.970

...((((((((........)).)))))) -16.60 6.035

...(((((((((......))).)))))) -18.70 6.164

...(((((((((......)).))))))) -18.70 6.276

...

...

...

......((.(.......).))....... -0.70 6359222.695

.....(((.(.......).)))...... -4.00 6359222.706

...(.(((.(.......).))))..... -3.50 6359222.724

...(.(((((.......))))))..... -10.50 6359223.016

...(.(((((.......))))).).... -10.60 6359223.417

..((.(((((.......))))).).).. -10.10 6359223.529

..((((((((.......))))))).).. -17.10 6359223.556

(.((((((((.......))))))).)). -16.60 6359223.967

(.((((((((.......)))))).))). -16.60 6359224.168

(.((((((((.......))))).)))). -16.60 6359224.193

(.(((((((((.....)))))).)))). -18.50 6359224.481

(.(((((((((.....))))).))))). -18.50 6359224.796

(.(((((((((.....)))).)))))). -18.50 6359224.824

..(((((((((.....)))).))))).. -19.00 6359225.276

.((((((((((.....)))).)))))). -22.30 6359225.595

.((((((((((.....))).))))))). -22.30 6359225.828

.((((((((((.....)).)))))))). -22.30 6359225.845

.((((((((((.....).))))))))). -22.30 6359225.931

.(((((((((((...)).))))))))). -22.00 6359226.658

.(((((((((((...).)))))))))). -22.00 6359227.013

((((((((((((...).))))))))))) -24.50 6359228.189

(((((((((((......))))))))))) -30.50 6359228.256

((((((((((((....)))))))))))) -32.80 6359230.801
